# Prevalence of the additional head of quadriceps femoris in the South Indian population: a cadaveric and radiological study

**DOI:** 10.1038/s41598-021-95374-z

**Published:** 2021-08-09

**Authors:** Femina Sam, Madhavi Kandagaddala, Ivan James Prithishkumar, Koyeli Mary Mahata, Mahasampath Gowri, Suganthy Rabi

**Affiliations:** 1 Department of Anatomy, Christian Medical College Vellore, The Tamil Nadu Dr. MGR Medical University, Chennai, India; 2 Department of Radiodiagnosis, Christian Medical College, Vellore, India; 3 College of Medicine, Mohammed Bin Rashid University of Medicine and Health Sciences, Dubai, UAE; 4 The Alfred Hospital, Melbourne, VIC 3004 Australia; 5 Department of Biostatistics, Christian Medical College, Vellore, India

**Keywords:** Developmental biology, Systems biology, Anatomy

## Abstract

Quadriceps femoris is an extensor muscle in the anterior compartment of thigh and is traditionally taught to be composed of four heads. Recently, there is an increased interest in the occurrence of an additional muscle head of quadriceps femoris. But scientific knowledge regarding its incidence is lacking in the South Indian population. This study was done to confirm the presence of the additional head by routine anatomic dissection and radiological imaging techniques. Forty-one formalin fixed human cadaveric lower limbs were dissected and the morphology of the additional head was noted. Retrospective analysis of 88 MRI images of patients was done. The additional muscle head was present in 43.9% of the cadaveric lower limbs and was consistently located between the vastus lateralis and vastus intermedius. It originated from variable portions of the greater trochanter, intertrochanteric line, lateral lip of linea aspera and lateral surface of the shaft of femur and inserted either as a muscle belly or as an aponeurosis into the vastus intermedius (55.6%), vastus lateralis (22.2%) or directly into the base of the patella. It received its vascular supply from branches of the lateral circumflex femoral artery and was innervated by branches from the posterior division of the femoral nerve. In addition, the additional muscle head was identified by MRI and its incidence was reported to be 30.68% for the first time in living subjects. The result of this study provides additional information in understanding the morphology of the quadriceps femoris muscle.

## Introduction

The quadriceps femoris is the powerful extensor muscle of the knee joint present in the anterior compartment of thigh. It is traditionally described to consist of four parts—the rectus femoris and the three vasti, the aponeuroses of which merge at the base of the patella to form the Quadriceps tendon^1^. In 1990, Willan et al. first described the presence of an additional fleshy head between the vastus lateralis (VL) and vastus intermedius (VI) in 27 of the 40 cadavers dissected (36%)^2^. After a period of quiescence, recent case reports and studies have rekindled an interest on the occurrence of the additional head of quadriceps. However, it is not clear whether it is a separate muscle or if it is a part of vastus intermedius or lateralis^3–5^. Though absence of the rectus femoris is reported in literature, variations in the arrangement of quadriceps femoris is not known to be common^6^. Grob et al. described this additional muscle belly to be located between the vastus lateralis and vastus intermedius by dissection and named it as the tensor of vastus intermedius. It originated from the anterolateral aspect of the greater trochanter and became a variable broad, flat tendon or aponeurosis before merging distally with the quadriceps tendon^7^. Identification of the additional head by radiological imaging is also scarcely reported. In 2016, Rajasekaran et al. studied ultrasonographic imaging of the anterior thigh and observed the distal tendinous portion of the additional muscle head in all 40 knees of 20 (100%) subjects^8^. The aim of the current study is to describe the occurrence, attachments, variations, and morphometry of the additional muscle head of the quadriceps femoris in the South Indian population through cadaveric dissection and radiologic imaging.

## Materials and methods

This study was done in the Department of Anatomy in collaboration with the Department of Radiodiagnosis of Christian Medical College, Vellore, India after obtaining ethical approval from the Institutional Review Board, Christian Medical College, Vellore, India.

### Cadaveric study

**Figure 1:**
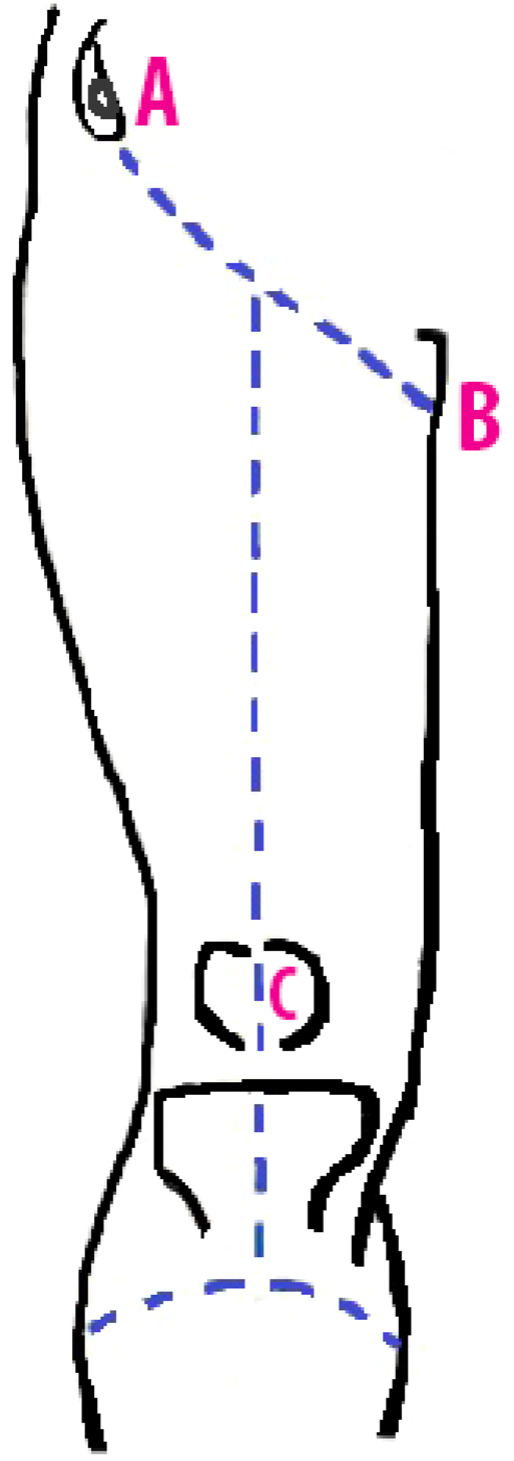
A horizontal incision was made on the thigh inferior and parallel to the inguinal ligament from the anterior superior iliac spine to the pubic tubercle (A–B). At the midpoint of the horizontal incision, a vertical incision was made till few centimetres below the apex of the patella (C).

A total of 41 lower limb cadaveric specimens including 22 males (9 paired and 4 unpaired) and 19 females (8 paired and 3 unpaired) belonging to the age group of 30–70 years were dissected, following the standard dissection protocol as described in the Cunningham Manual of Practical Anatomy^9^. These cadavers were obtained through the body donation programmes conducted by DC Body donation, Vellore Smart City Lions Club of Vellore and Udhavum Ullangal, Vellore after getting informed consent from the relatives while donating. These cadavers were donated for the purpose of teaching and research purposes. The cadavers were embalmed with formalin. Cadavers with disfigurement or injury in the femoral region were excluded. A horizontal skin incision was made on the anterior thigh immediately inferior and parallel to the inguinal ligament. At the midpoint of the horizontal incision, a vertical incision was made till few centimetres below the apex of the patella, where a second horizontal was made (Fig. 1).

After reflecting the skin, superficial fascia and deep fascia, the muscles of the anterior compartment of the thigh were exposed. The presence of an additional muscle head in between the vastus lateralis and vastus intermedius was sought by retracting vastus lateralis laterally. The shape of the muscle was noted. The length, breadth and the thickness of the muscle belly, the length and the breadth of the aponeurosis and the distance between the fusion of aponeurosis of the additional muscle head with the aponeurosis of quadriceps femoris to the insertion into the patella were measured using an inch tape and a digital vernier calliper. Branches of the femoral artery and the femoral nerve supplying the muscle bellies were traced. All the dissected specimens were photographed using a digital camera.

### Radiological study

High resolution MRI scans was initially done on a sample of 12 cadaveric lower limbs using a 3-Tesla Philips Achieva system to investigate the occurrence and appearance of the additional head of quadriceps femoris and followed by routine anatomical dissection to confirm its presence or absence. The MRI images were acquired in axial and coronal planes using T1 and T2 sequences. After confirming the presence of the additional muscle head in cadaveric MRI, a further retrospective study of 102 MRI images (both lower limbs of 16 males and 35 females) were studied of which the initial 14 MR images were used for sample size determination. Lower limbs with any structural abnormalities, fractures or hematoma involving the femur or muscular atrophy with fatty degeneration were excluded. Since fourteen MR images were already used for the pilot study and sample size estimation, the data was analysed for 88 MR images.

Data was entered in Microsoft excel and statistical analysis was performed using STATA V.13.1.

### Ethical approval

This study was in accordance with the ethical standards of the Institutional Review Board of Christian Medical College, Vellore, India.

## Results

### Cadaveric study

**Figure 2:**
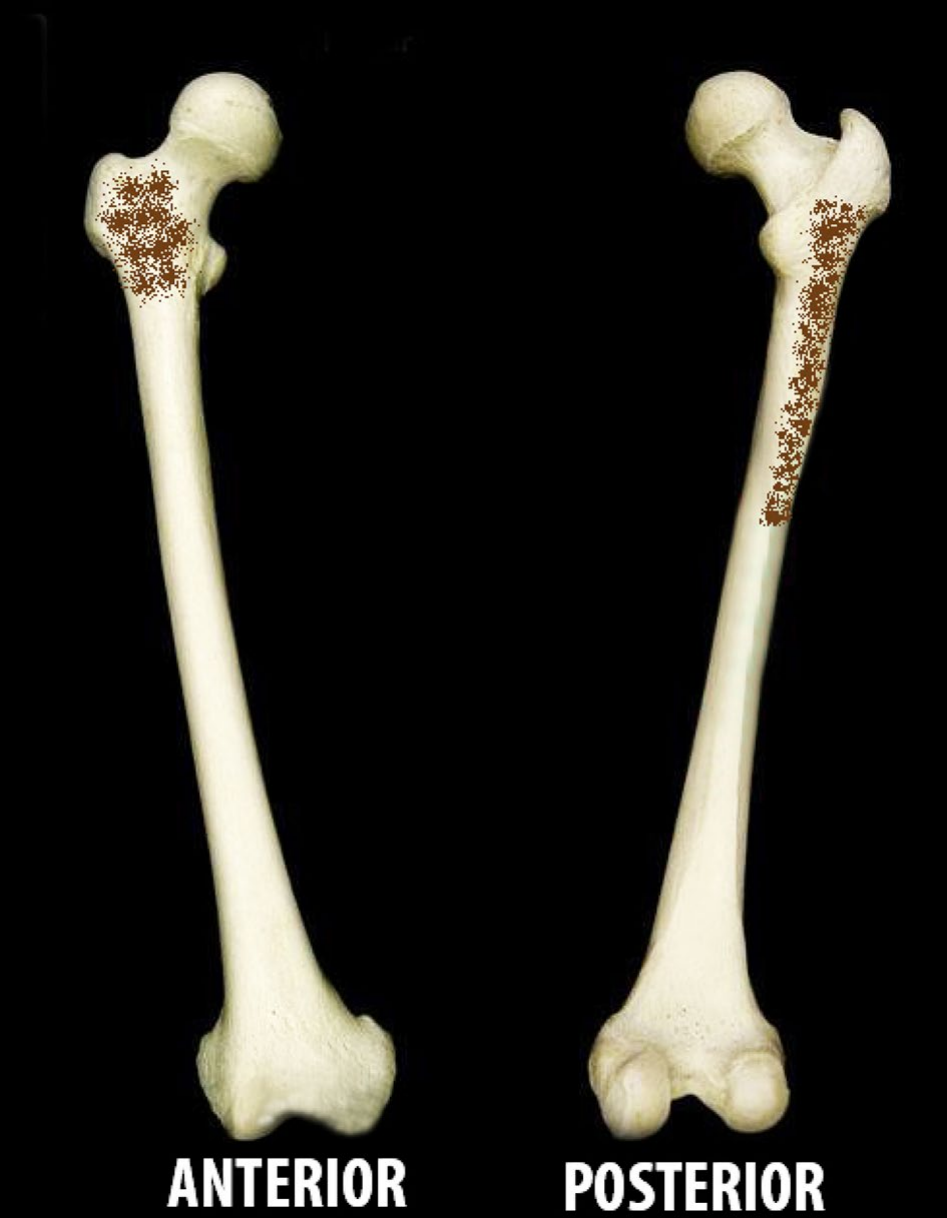
Schematic diagram showing the origin of additional muscle head of quadriceps femoris.

**Figure 3:**
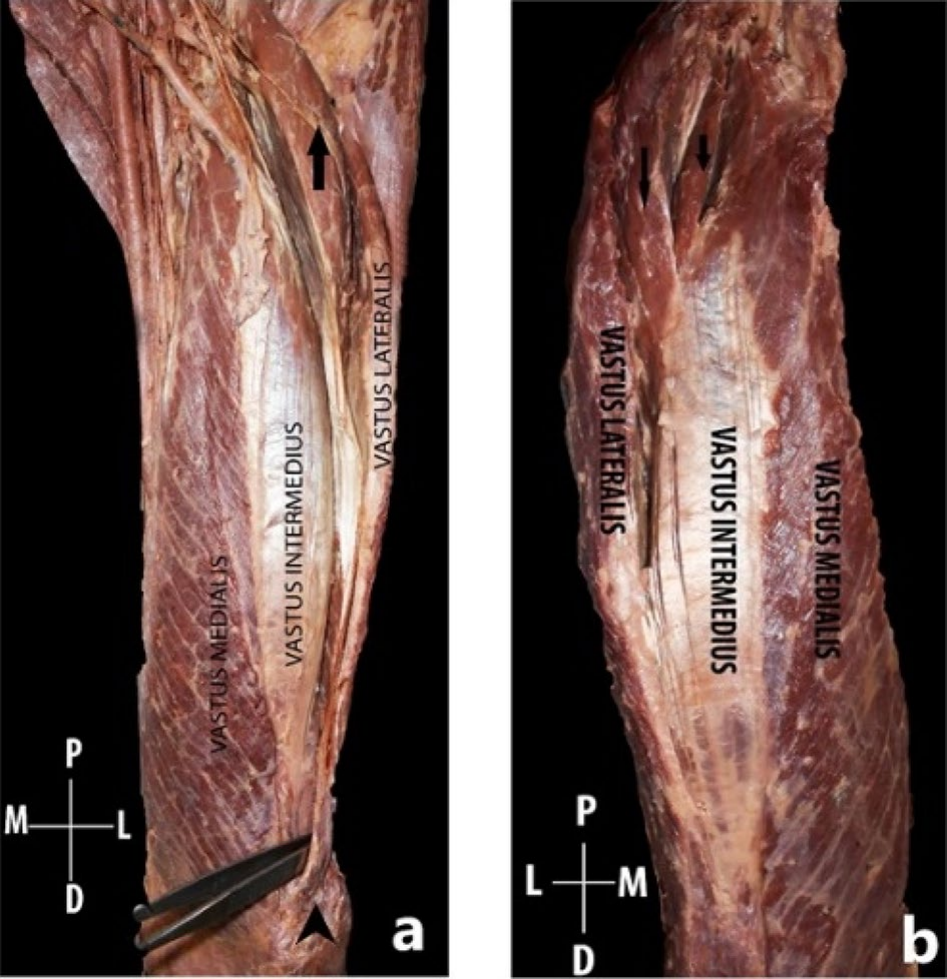
The insertion of the additional muscle head directly into the base of patella which is represented by a black arrowhead (**a**), the insertion of the additional muscle head into the aponeurosis of vastus intermedius (**b**). The black arrow shows the additional muscle head.

**Figure 4:**
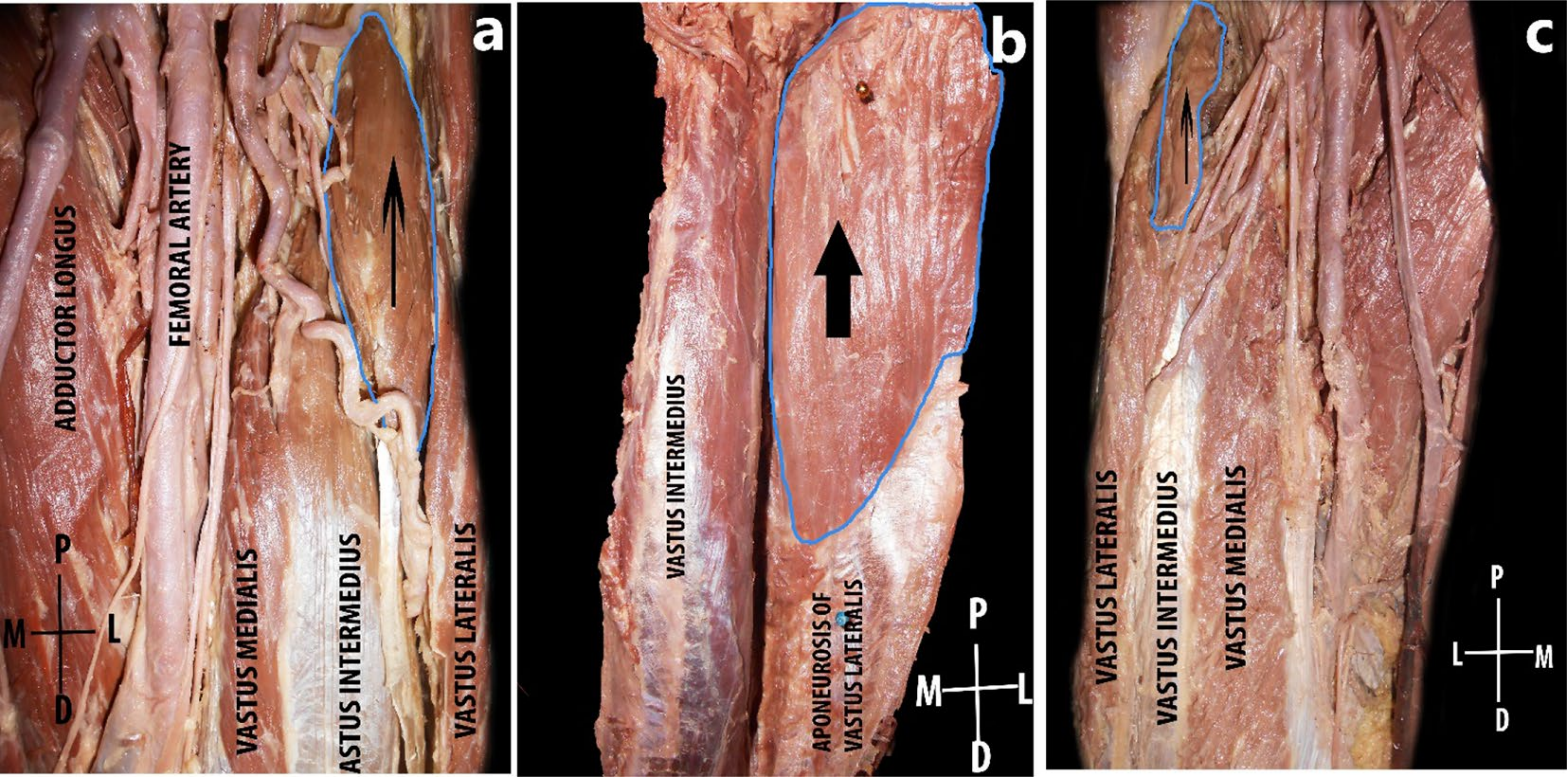
Anterior aspect of the thigh; The rectus femoris muscle has been reflected. The additional muscle head depicted in arrows, presented in different shapes like fusiform (**a**), quadrilateral (**b**), and slender (**c**).

In a total of 41 lower limbs dissected, the additional muscle head of quadriceps femoris was present in 43.9% (45.45% in males and 42.10% in females) of limbs. There was no significant gender discrepancy (*p* = 0.497) and though the additional head was present more on the left side compared to right, it was statistically insignificant (*p* value = 0.829). In all the cases, the additional muscle head was located in between vastus lateralis and vastus intermedius. It took origin either from the greater trochanter, intertrochanteric line, lateral lip of linea aspera or upper one fourth of the lateral surface of the shaft of femur. The additional head was inserted into the vastus intermedius (55.56%) or vastus lateralis (22.22%) as an aponeurosis or directly as a muscle belly (Figs. 2, 3). In one lower limb, the additional head had a conjoint origin along with vastus lateralis and in two lower limbs the aponeurosis of the additional head directly inserted into the base of the patella along with the quadriceps tendon. It occurred as a single muscle belly (77.7%), two muscle bellies (16.6%) or three muscle bellies (5.55%) and its shape was also quite variable, being predominantly fusiform in shape followed by quadrilateral and slender shaped (Fig. 4). There was no statistically significant difference between males and females in its morphometry such as length, breadth additional muscle head of quadriceps femoris.

**Figure 5:**
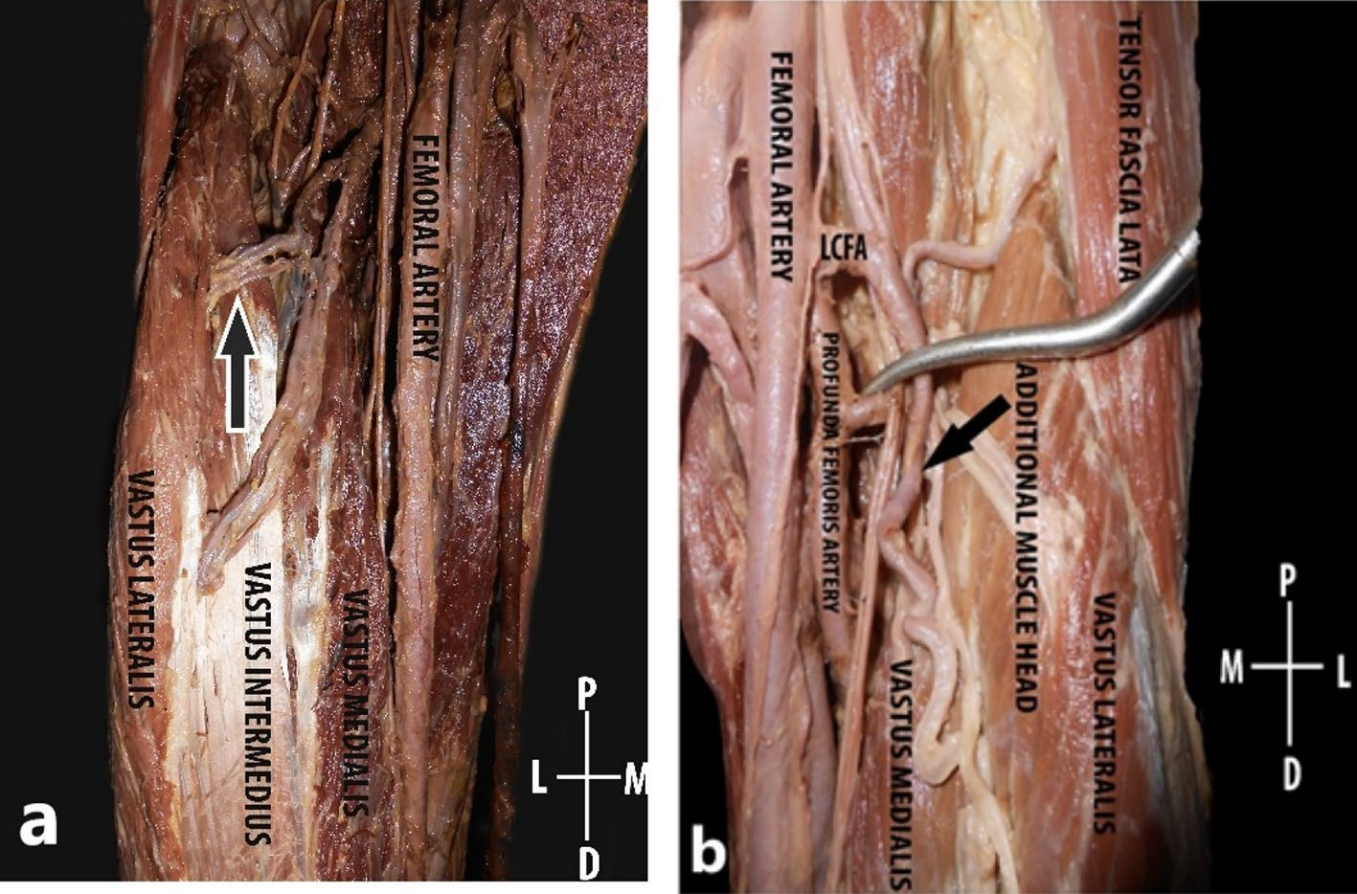
Anterior aspect of proximal third of thigh. The rectus femoris muscle has been reflected. The arrow shows the additional muscle head getting its blood supply from the transverse branch of LCFA (**a**). Arrow indicating the direct branch of profunda femoris artery and the descending branch of LCFA was having a highly tortuous course (**b**).

The additional muscle head got its vascular supply either from the descending or transverse branches of the lateral circumflex femoral artery (LCFA) (Fig. 5a). In one lower limb, the additional muscle head was supplied by a direct branch of the profunda femoris artery and in that lower limb, the descending branch of lateral circumflex femoral artery had a highly tortuous course in the anterior aspect of the thigh in between the additional muscle head of quadriceps femoris and the vastus medialis (Fig. 5b).

**Figure 6:**
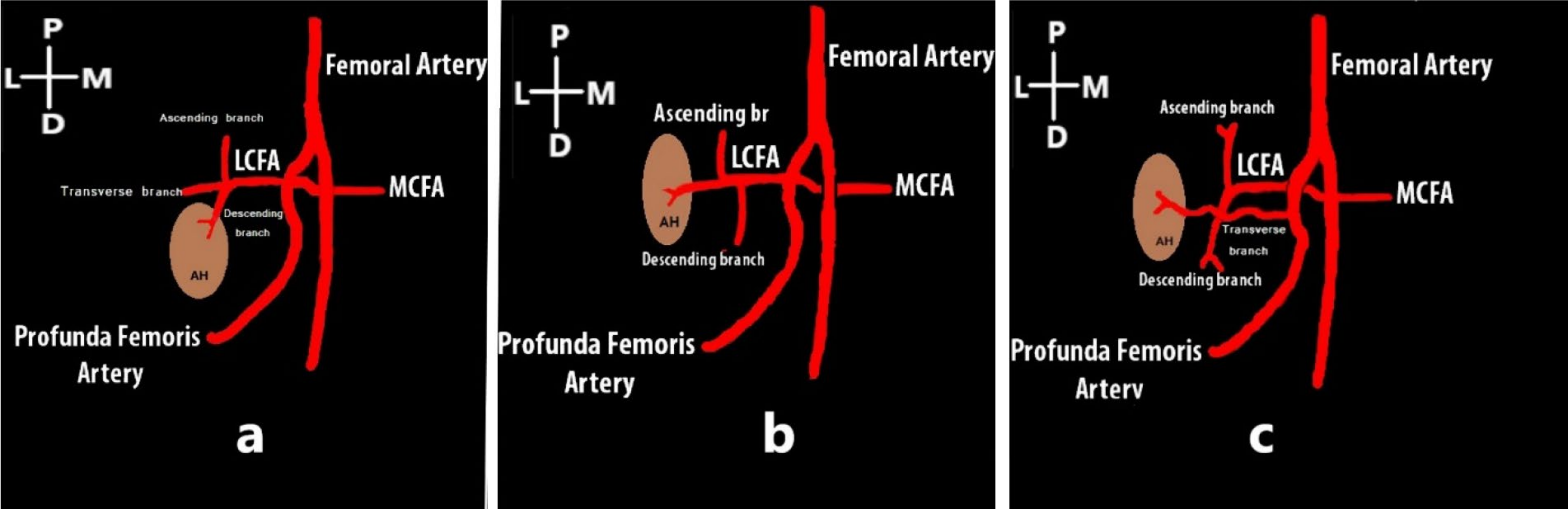
Schematic diagram showing the arterial supply to the additional muscle head; LCFA—lateral circumflex femoral artery, MCFA—medial circumflex femoral artery, AH—additional muscle head.

In the present study, the vascular supply of the additional muscle head was classified into three types (Fig. 6):

- **Type 1**—descending branches of the lateral circumflex femoral artery
- **Type 2**—transverse branches of the lateral circumflex femoral artery
- **Type 3**—direct branch of the profunda femoris of femoral artery

**Figure 7:**
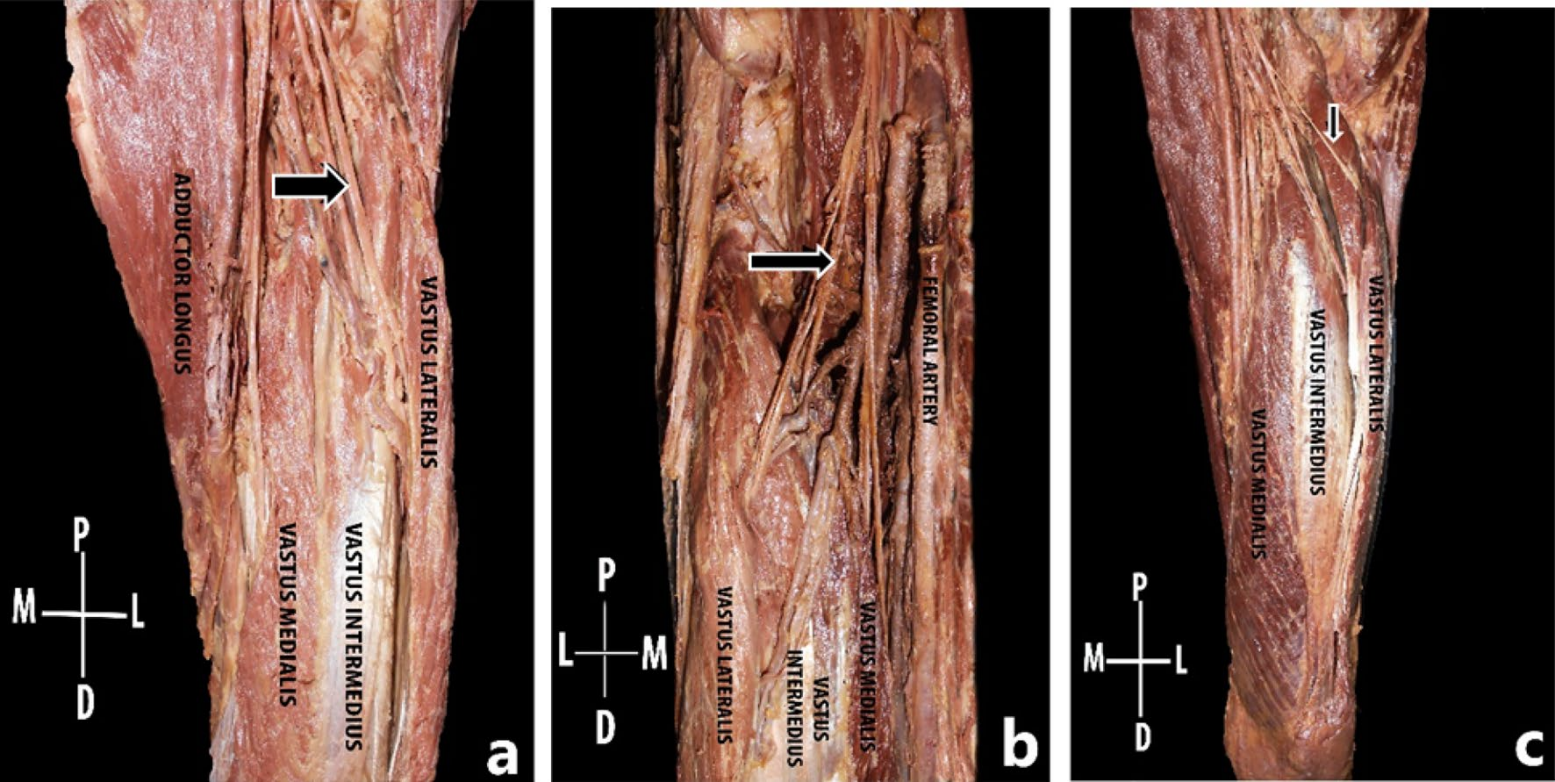
Anterior aspect of the thigh; The arrows indicating the nerve supply of the additional muscle head; by the direct branch of femoral nerve (**a**), by the nerve to vastus lateralis (**b**) and by the nerve to vastus intermedius (**c**).

The additional muscle belly was innervated either by a direct branch of the posterior division of femoral nerve or from the nerve to vastus lateralis or the nerve to vastus intermedius (Fig. 7).

**Figure 8:**
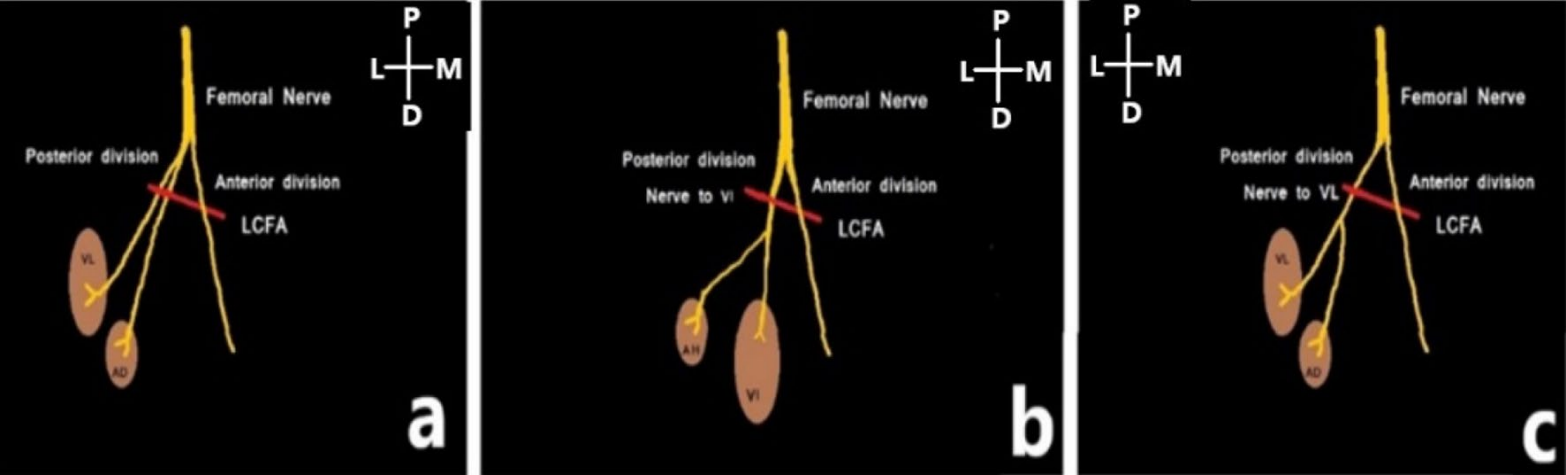
Schematic diagram showing the nerve supply to the additional muscle head; LCFA- lateral circumflex femoral artery.

In the present study, the nerve supply of the additional muscle head was classified as follows (Fig. 8):

- **Type 1**—the direct branch of posterior division of femoral nerve
- **Type 2**—from the nerve to vastus lateralis
- **Type 3**—from nerve to vastus intermedius

### Radiological study

High resolution MRI scans of 12 cadaveric lower limbs showed the presence of the additional muscle head in 4 lower limbs. Dissection of these 12 lower limbs revealed the presence of additional head in all the 4 limbs. Hence, the concordance rate of dissection and MRI study was 100%.

**Figure 9:**
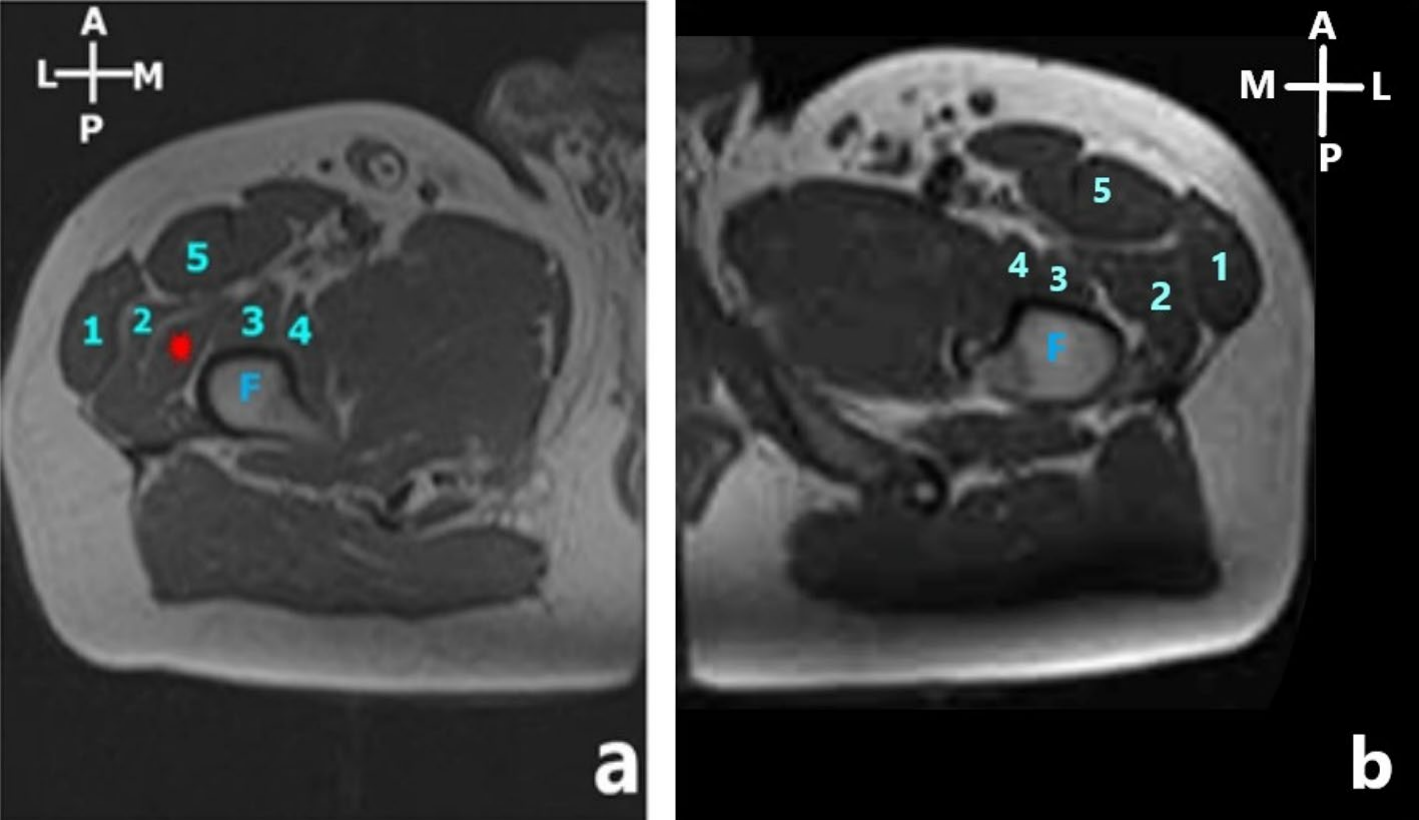
T1 weighted axial MR imaging of the lower limb of a patient; 1—tensor fascia lata, 2—vastus lateralis, 3—vastus intermedius, 4—vastus medialis, 5—rectus femoris, F—femur. The additional muscle head () is depicted in between the vastus lateralis and vastus intermedius (**a**) and absence of additional muscle head is depicted in (**b**).

Retrospective MRI images of the lower limbs obtained from 44 patients (28 female and 16 male) showed the definitive presence of the additional head in 30.68% (37.5% in males and 26.7% in females) of individuals (Fig. 9).

## Discussion

Variations in the anterior muscle group of thighs are not common. Yet, Willan et al. (1990) identified for the first time, an additional fleshy belly between the vastus lateralis and vastus intermedius in 27 of the 40 cadavers dissected (36%), the fleshy belly being bilateral in 10 subjects and unilateral in seven^2^. In 2016, Grob et al. published a landmark article highlighting the re-discovery of this additional head. Since then, there have been few case reports and studies which describe its occurrence. The occurrence of these additional heads has also raised speculations on whether the quadriceps should be called as multiceps femoris^10^. It was Grob et al. who had initially termed the additional head as a ‘tensor of the vastus intermedius’ and this term has crept into most of the subsequent publications^11^. Both Grob et al. and Veeramani et al. report the prevalence of the additional head in 100% of individuals studied. Both report the position of the fleshy head to be located between the vastus lateralis and vastus intermedius^11, 12^. However, other studies by Collins et al. and Alimohammed report a lower prevalence^4, 13^. The present study reports the occurrence of the additional head in about half of the subjects studied (44%) and it was located between the VL and VI in all these subjects.

Regarding the origin of the additional muscle head, Grob et al. report that the muscle bellies of vastus lateralis, the tensor of vastus intermedius, and the vastus intermedius present a common, hardly-divisible origin between the intertrochanteric line and greater trochanter^3^. In the present study, the additional head took origin from the greater trochanter of the femur, or from greater trochanter and lateral lip of linea aspera, or from the intertrochanteric line and lateral surface of the shaft of the femur. The additional head is reported to have between 1 and 3 bellies, the most common being 2 bellies which is similar to our findings in 16.6% of subjects^3, 12^. The shape of the additional muscle belly had not been described before. The muscle bellies of the additional head were predominantly fusiform in shape followed by quadrilateral and slender shaped. The mean length of the additional head belly was 116.5 ± 28.0 mm and is significantly smaller to the mean length reported by Veeramani et al. (female: 162.6 ± 47.4 mm; male: 139.7 ± 32.7 mm)^12^.However, there was no gender difference. No other studies have mentioned the breadth and thickness of the additional muscle head. Veeramani et al. also report the mean length of the aponeurosis of the additional head to be 193.55 ± 42.32 mm with a significantly higher value in females compared to males^12^. However, the length of the aponeurosis was found to be much smaller with no significant gender difference (Table 1).

**Table 1:** Comparison of the mean of variables in cadaveric males and females.

Variables	Minimum (cm)	Maximum (cm)	Mean (cm)			*p* value
			Overall	Males	Females	
Length of the muscle	7	15	11.65 ± 2.80	11.49 ± 2.90	11.84 ± 2.86	0.80
Breadth of the muscle	2.17	11.50	5.99 ± 2.63	5.98 ± 2.52	6.00 ± 2.86	0.98
Width of the muscle	0.90	7.50	2.88 ± 1.95	2.43 ± 1.40	3.43 ± 2.47	0.29
Length of the aponeurosis	2.00	27.50	22.20 ± 7.71	22.00 ± 9.92	22.50 ± 3.76	NA
Breadth of the aponeurosis	1.50	7.50	5.85 ± 2.07	5.33 ± 2.48	6.63 ± 1.11	NA
Aponeurosis of additional muscle to the base of patella	8.00	27.00	22.25 ± 4.21	23.45 ± 2.20	20.75 ± 5.67	0.18

Most earlier studies have mentioned that the additional muscle head consistently had an aponeurosis which inserted into the base of patella^3, 12^. However, in 44.4% of the lower limbs, the additional muscle head did not have any aponeurosis. This finding has not yet been described in any other previous studies. Grob et al. previously classified the additional head of the quadriceps femoris into sub-types based on the interaction of the aponeurosis with the vastus lateralis and vastus intermedius, into an independent-type (most common), vastus intermedius type, vastus lateralis type and a common type^3^. Alimohammadi reported an almost equal proportion of the independent and vastus intermedius type, whereas both Grob et al. and Veeramani et al. found a predominance of the independent type^3, 4, 12^. In contrast to these reports, the most common type noticed in the present study was the vastus intermedius type (55.56%) where the additional muscle head inserted to the aponeurosis of the vastus intermedius, followed by the vastus lateralis type (22.22%) and then by the independent type which was present in only 11.1% of lower limbs. Hence, we propose that this fleshy belly may be considered only as an additional head of the quadriceps femoris, and not as an individual muscle.

**Figure 10:**
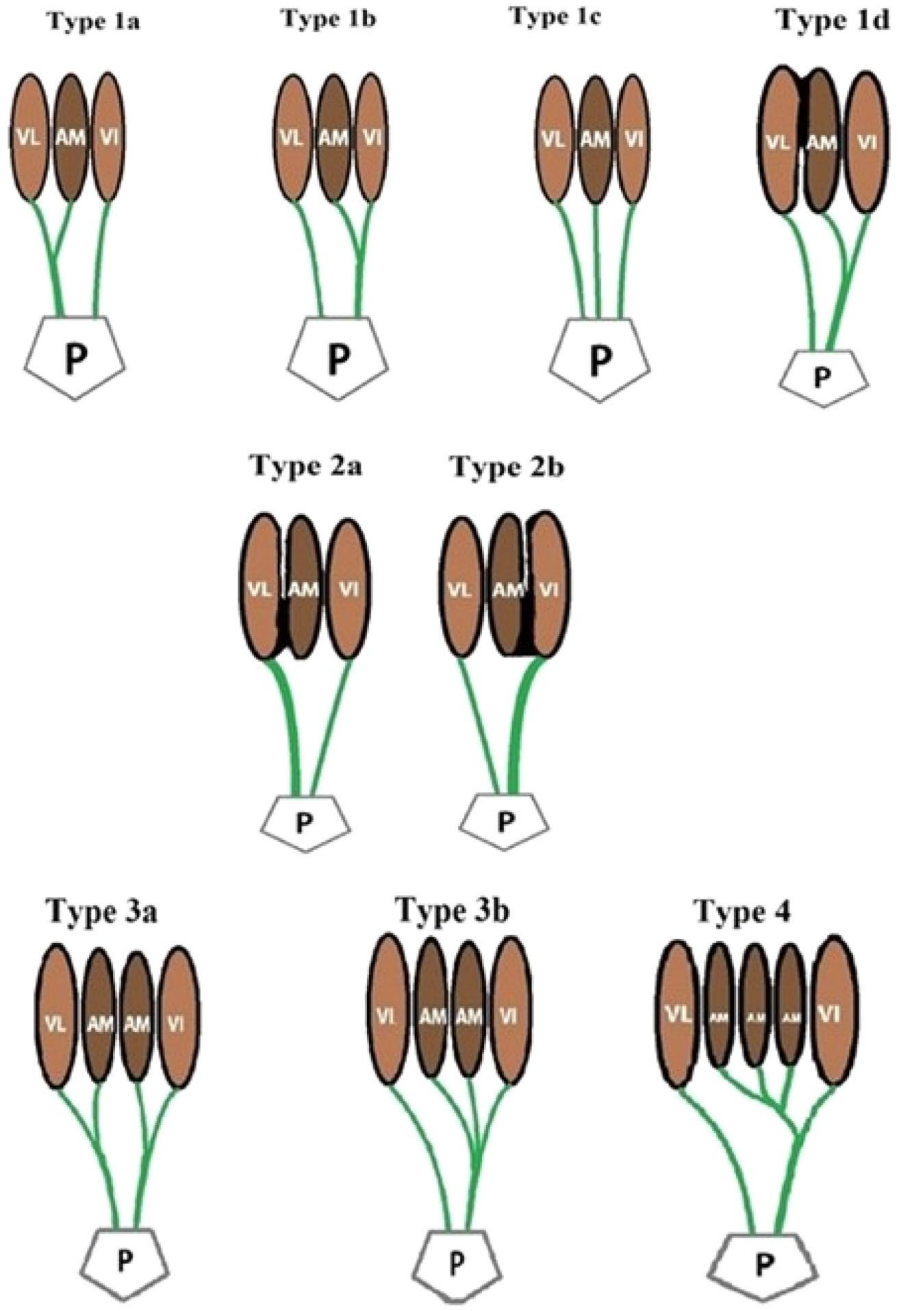
Morphological types of additional head of quadriceps femoris muscle; VL—vastus lateralis, VI—vastus intermedius, VM—vastus medialis, AM—additional muscle head; P—Patella.

Based on the findings of the present study, the morphology of the additional head can be classified as follows (Fig. 10):

**Type 1: Single muscle head with aponeurosis**

- Type 1 a: Independent origin and merges with the vastus lateralis
- Type 1 b: Independent origin and merges with the vastus intermedius
- Type 1 c: Independent origin and a separate insertion to patella
- Type 1d: Fused proximally with the vastus lateralis but distally the aponeurosis merges with the vastus intermedius

**Type 2: Single muscle head with no aponeurosis**

- Type 2a: Fused with the vastus lateralis
- Type 2b: Fused with the vastus intermedius

**Type 3: Two muscle bellies with aponeurosis**

- Type 3a: One muscle belly merge with the vastus lateralis and other to the vastus intermedius.
- Type 3b: Both muscle bellies merges with the vastus intermedius

**Type 4: Three muscle bellies merge with the vastus intermedius**

The additional muscle head has been reported to receive arterial supply directly from the lateral circumflex femoral artery or through individual branches of the transverse or ascending branches of the lateral circumflex femoral artery^3, 12^. In the present study, the additional muscle head was supplied by the transverse or descending branch of the lateral circumflex femoral artery. In only one case, the additional head was supplied by direct branches of the profunda femoris artery. But the additional head did not receive any vascular supply from the ascending branch of lateral circumflex femoral artery in contrast to the study done by Grob et al. Anatomically, the aponeurosis of the tensor of vastus intermedius runs adjacent to the descending branch of the lateral circumflex femoral artery. Therefore, injury to the tensor of vastus intermedius can cause rupture of the adjacent vessels resulting in haemorrhage and formation of a hematoma.

The additional muscle head was innervated by a direct branch from the posterior division of femoral nerve, or by branches from the nerve to vastus lateralis or nerve to vastus intermedius.

To understand the role of the additional head of quadriceps femoris in a better manner, knowledge of its gross morphology as well as its radiological features needs to be studied^14^. Recently, Rajasekaran et al. in 2016 reviewed the ultrasonographic appearance of the anterior thigh in 20 subjects and observed the distal tendinous portion of the tensor of vastus intermedius in all 40 knees of the 20 subjects^8^. Although sonography is generally accepted as a useful means to diagnose quadriceps tendon injury, its reliability has been questioned. MRI has been considered more accurate in assessing the extent of soft tissue and tendon injuries^15^. In the present study, the concordance rate of observing the additional head in cadaveric dissection following MRI was 100% concluding that MR imaging of the additional muscle head has high sensitivity and specificity. The additional muscle head has been demonstrated by MRI and it was present in 30.68% of the MR images of patients analysed retrospectively. However, the present study differs from the study done by Rajasekaran et al. who demonstrated the presence of the additional head in 100% of subjects studied by ultrasonography. There are very few reports of clinical correlations of the additional head^16, 17^. Labbe et al. in 2011 presented a case of symptomatic, progressive restriction of knee flexion in a nine-year-old girl in whom MRI and ultrasonography revealed the unusual presence of an accessory head of quadriceps femoris^16^. Further electromyographic studies of the additional head may reveal functional information on the knee and patellar biomechanics in these subjects. The presence of the additional muscle head may be responsible for undiagnosed knee pain and altered biomechanics.

## Conclusion

In conclusion, this is the first study to describe the appearance, location, and incidence of the additional muscle head of quadriceps femoris by MR imaging. MRI has high sensitivity and specificity for detecting the additional head. In the present study, the most common type was the vastus intermedius type where the additional muscle head inserted into the aponeurosis of the vastus intermedius (55.56%). Further scientific work should be done to investigate the role of the additional head in the biomechanics of the knee joint, patellar movement, joint stability, and the correlation of its occurrence to an individual’s lifestyle.
